# Supplementary material for: Differential responses of Cacao pathogens Colletotrichum gloeosporioides and Pestalotiopsis sp. to UVB 305 nm and UVC 275 nm
Source: Sci Rep. 2025 Oct 16;15:36256. doi: 10.1038/s41598-025-20277-2 (PMC12533136; doi:10.1038/s41598-025-20277-2)
Supplement: Supplementary file 1 — Supplementary Material 1 [file 41598_2025_20277_MOESM1_ESM.pptx]

## Slide 1
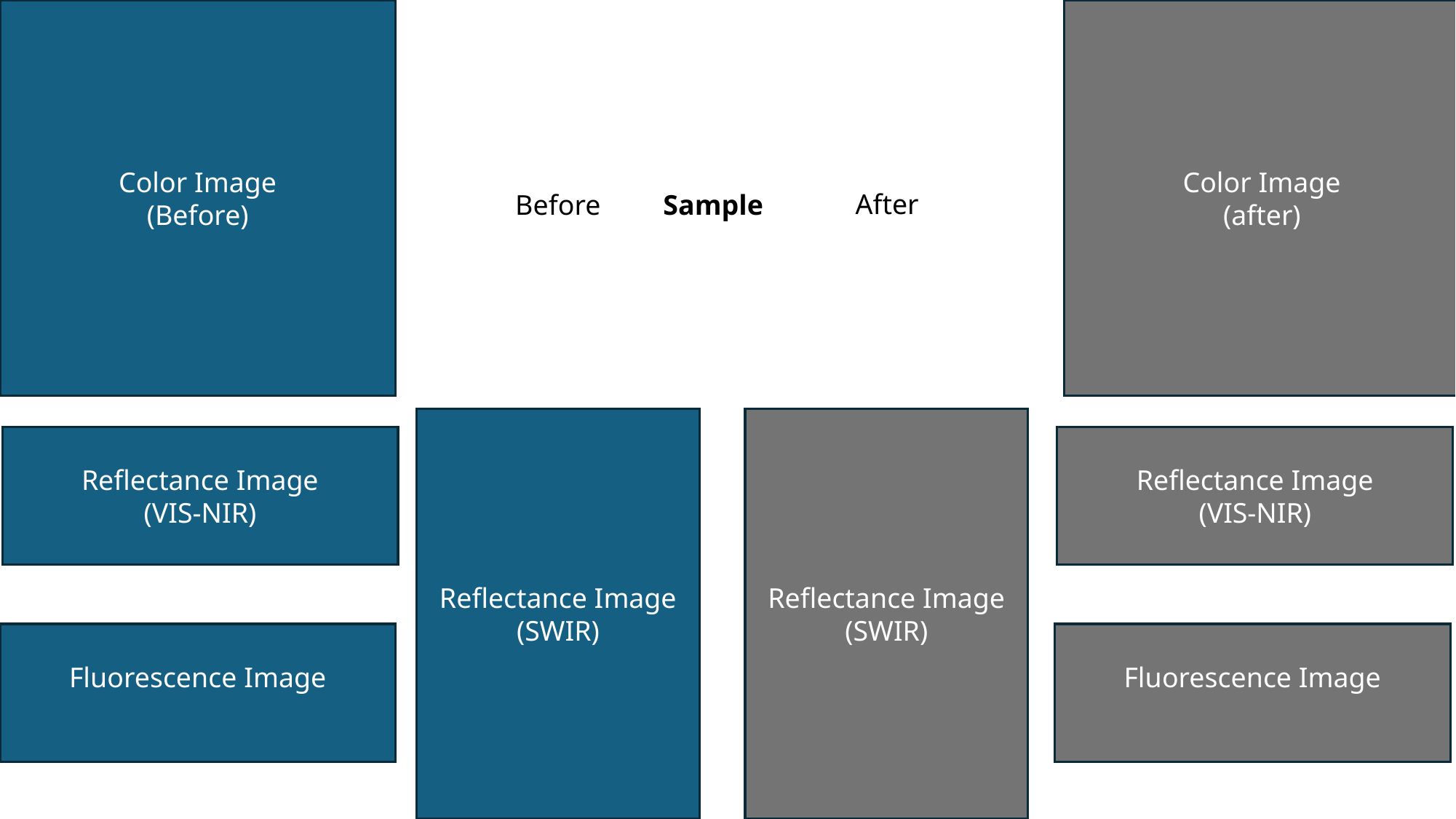

Color Image
(Before)
Color Image
(after)
After
Before
Sample
Reflectance Image
(SWIR)
Reflectance Image
(SWIR)
Reflectance Image
(VIS-NIR)
Reflectance Image
(VIS-NIR)
3
5
3
5
5 3
5 3
5 3
Fluorescence Image
Fluorescence Image
5 3
5 3

## Slide 2
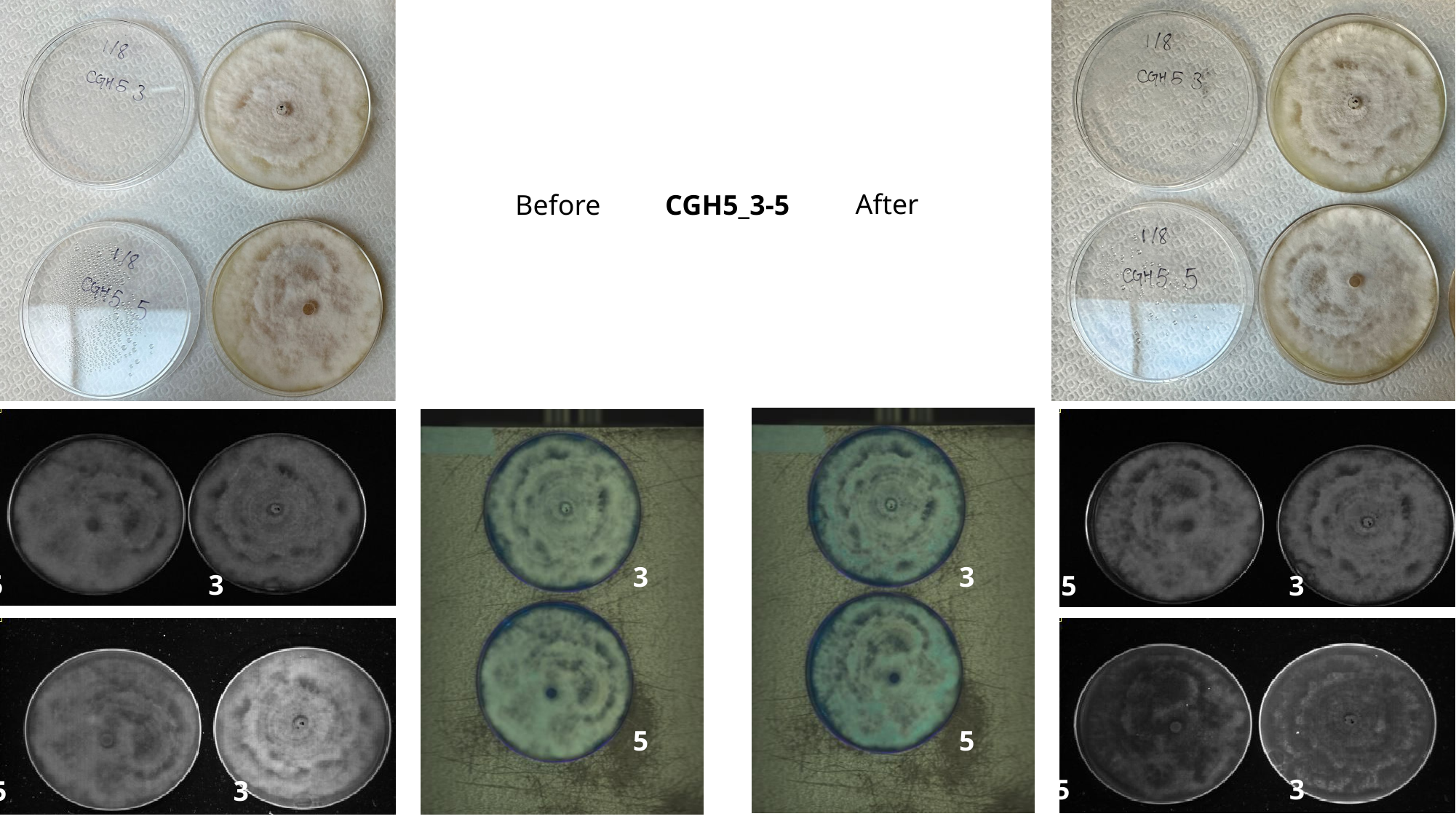

After
Before
CGH5_3-5
3
5
3
5
5 3
5 3
5 3
5 3
5 3

## Slide 3
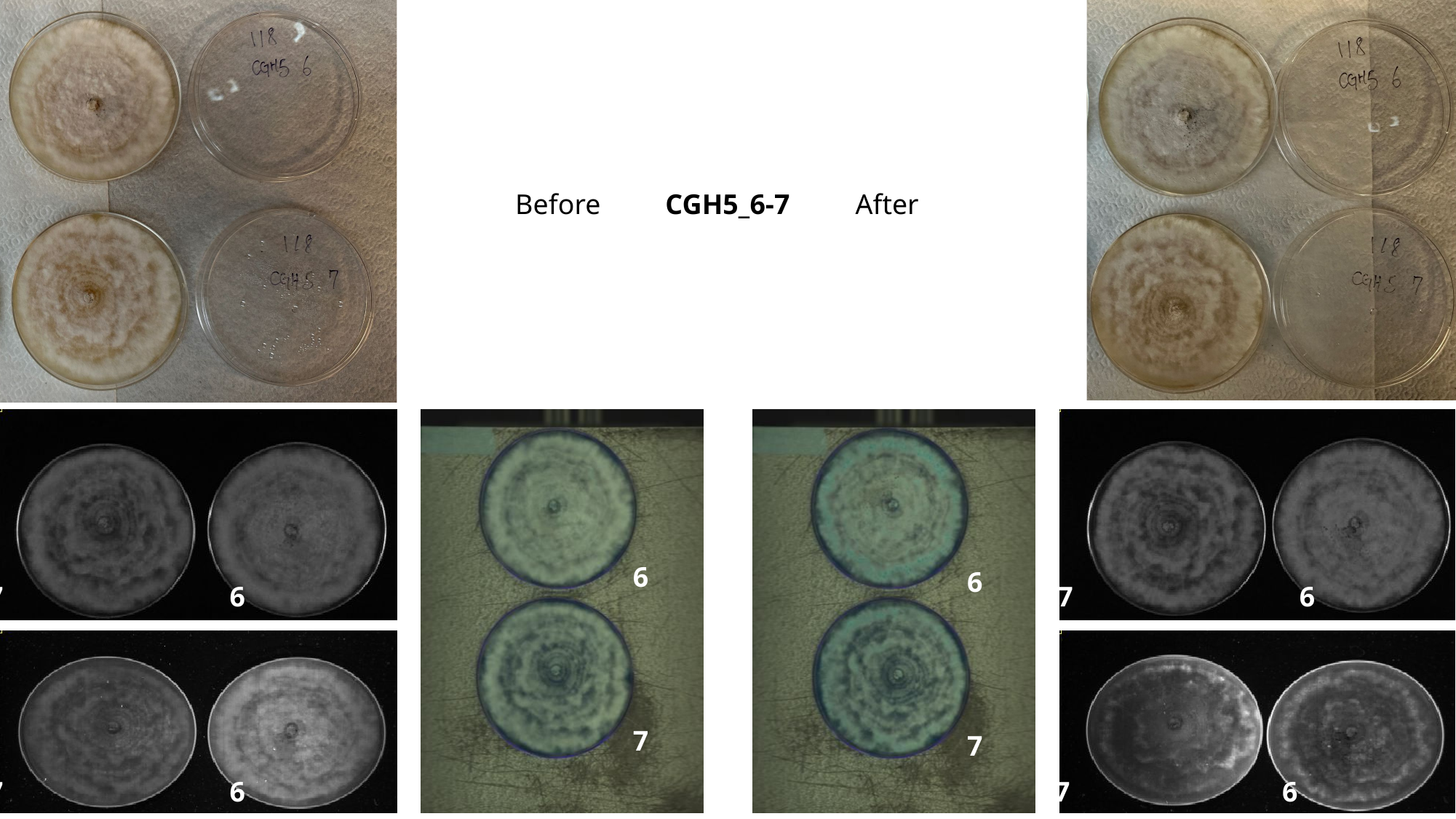

Before
CGH5_6-7
After
6
7
6
7
7 6
7 6
7 6
7 6

## Slide 4
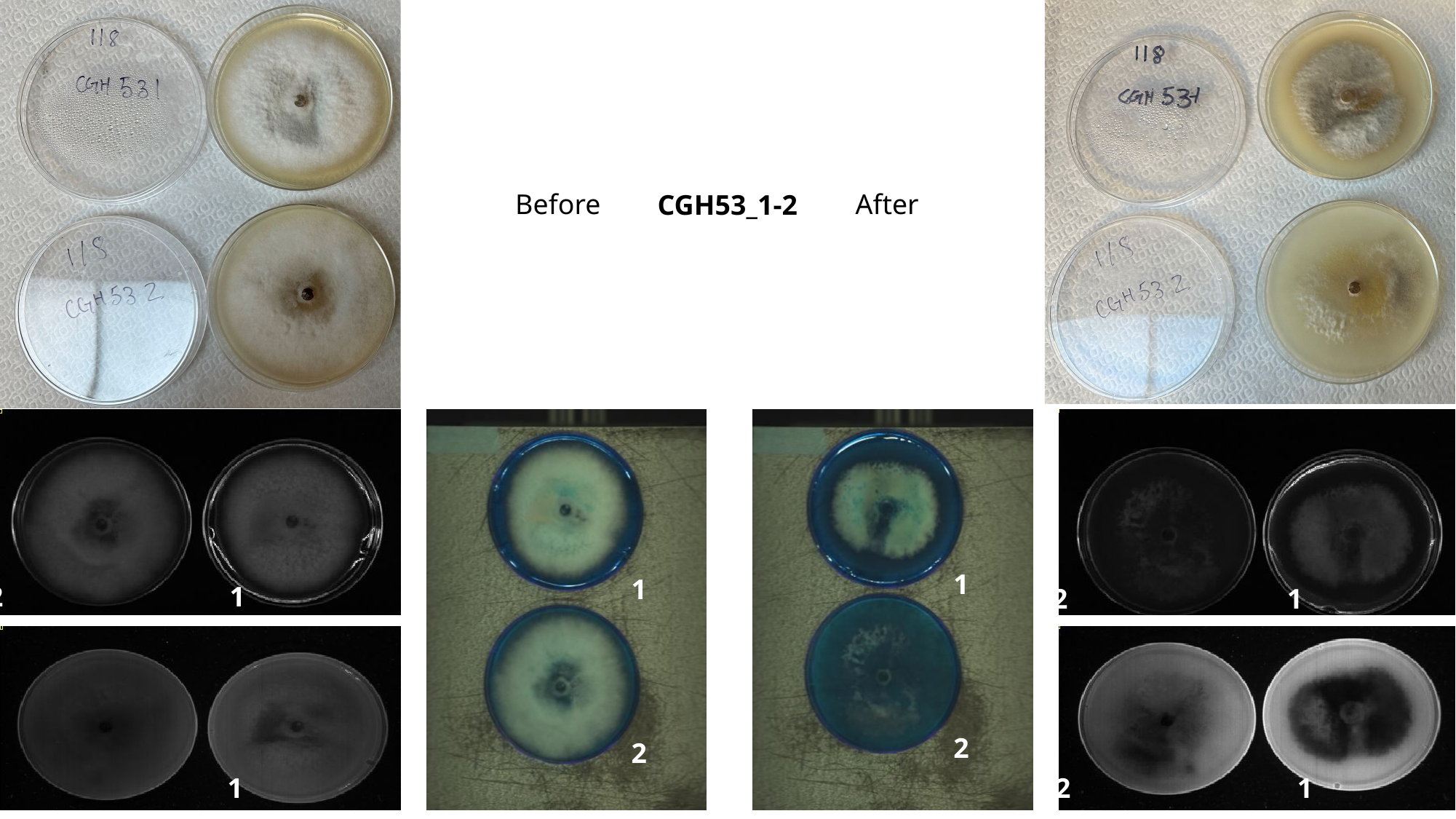

Before
After
CGH53_1-2
1
2
1
2
2 1
2 1
2 1
2 1

## Slide 5
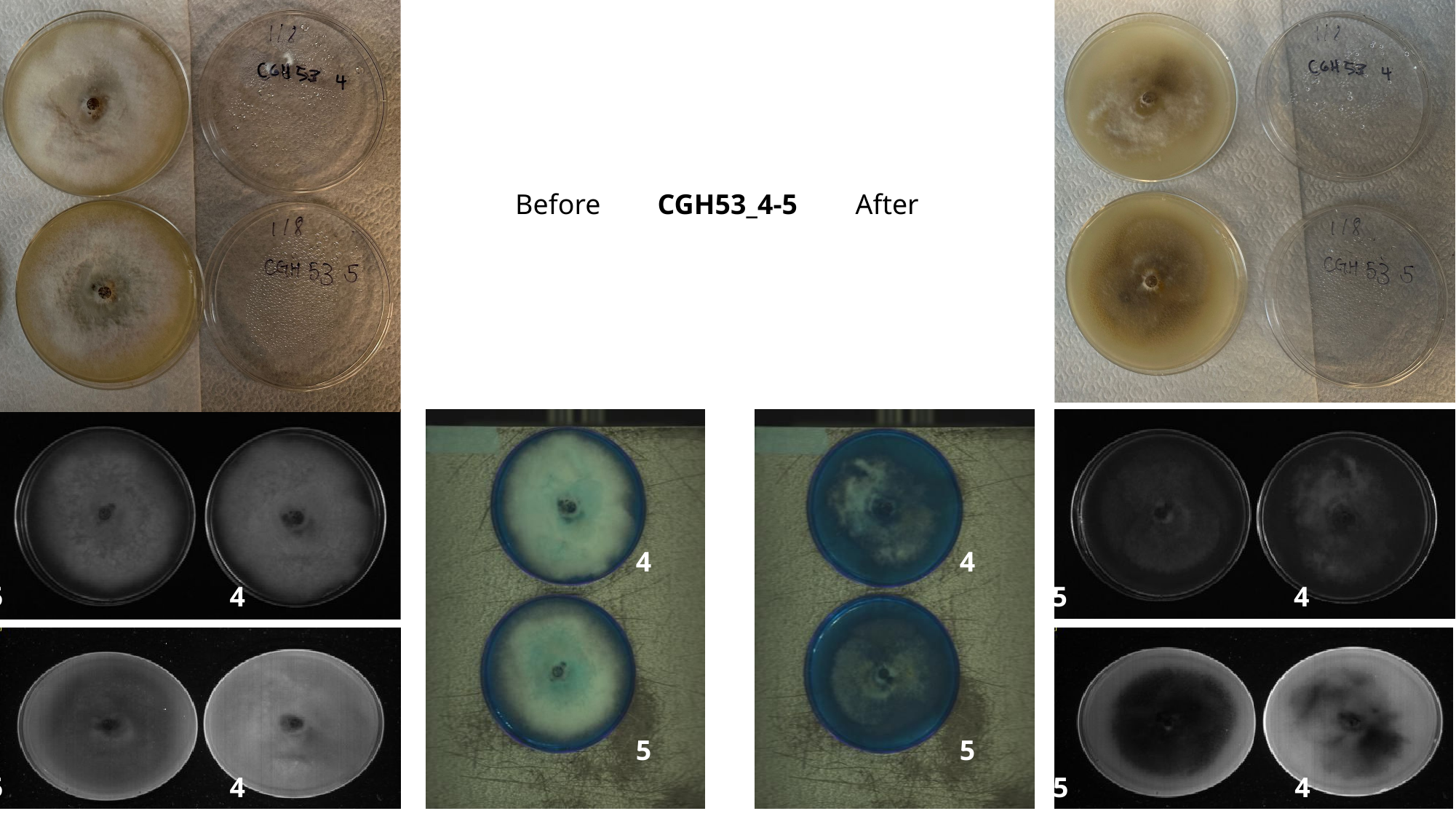

Before
CGH53_4-5
After
4
5
4
5
5 4
5 4
5 4
5 4
