# Supplementary figures and images for: Differential responses of Cacao pathogens Colletotrichum gloeosporioides and Pestalotiopsis sp. to UVB 305 nm and UVC 275 nm
Source: Sci Rep. 2025 Oct 16;15:36256. doi: 10.1038/s41598-025-20277-2 (PMC12533136; doi:10.1038/s41598-025-20277-2)

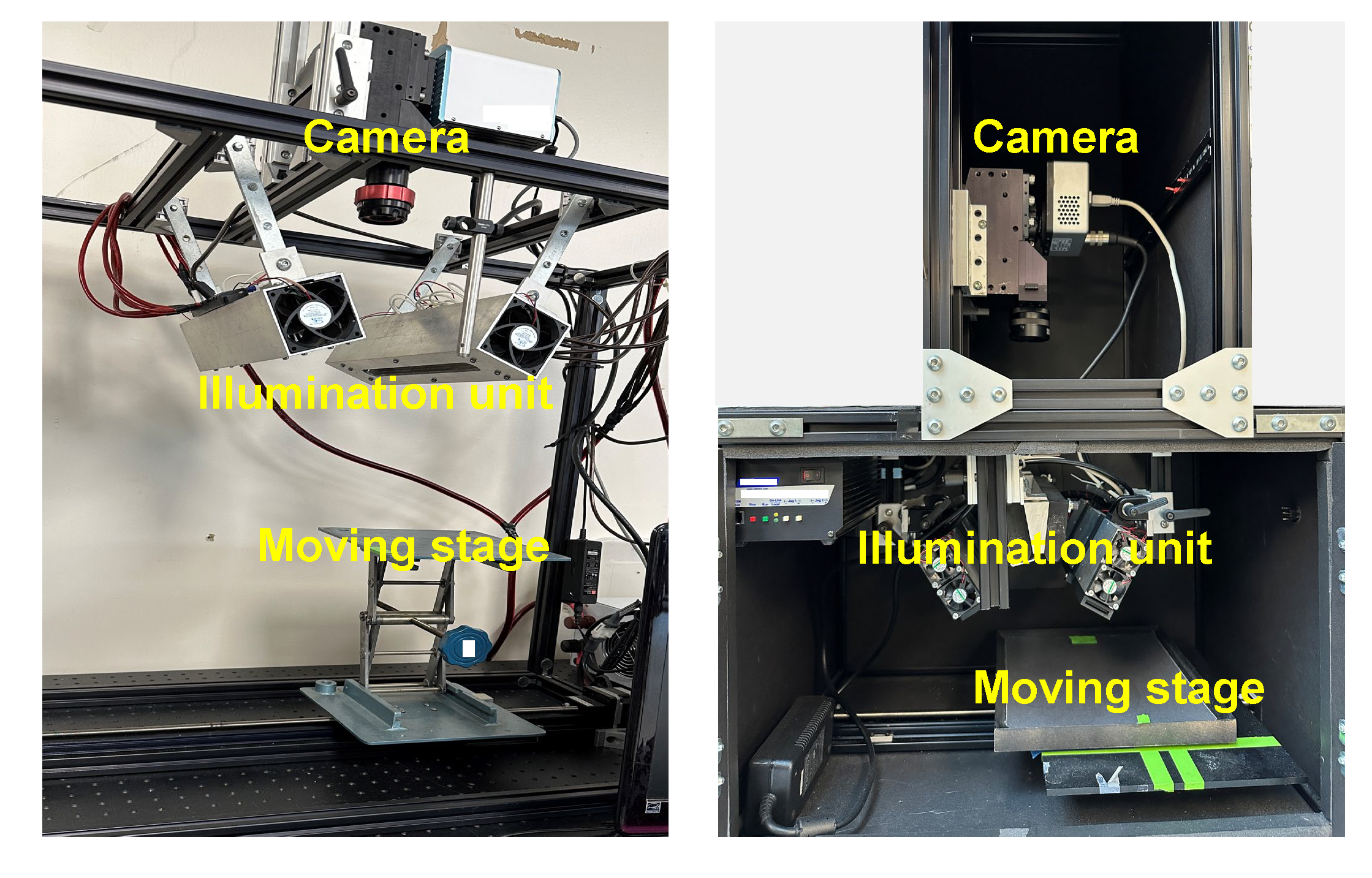

Supplement: Supplementary file 4 — Supplementary Material 4 [file 41598_2025_20277_MOESM4_ESM.zip › Fig. S5.-logo deleted.tif]

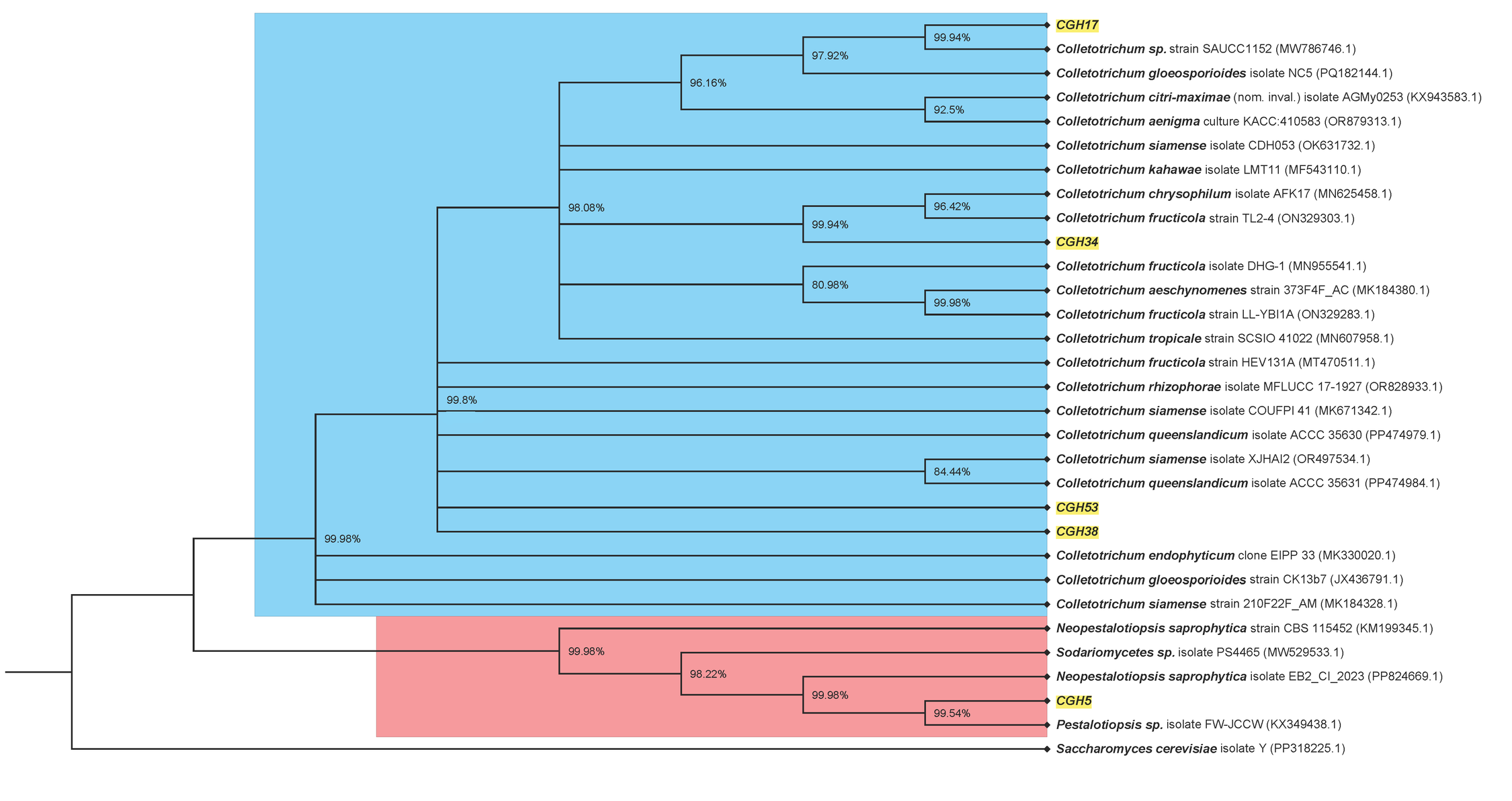

Supplement: Supplementary file 4 — Supplementary Material 4 [file 41598_2025_20277_MOESM4_ESM.zip › Fig. S1..tif]

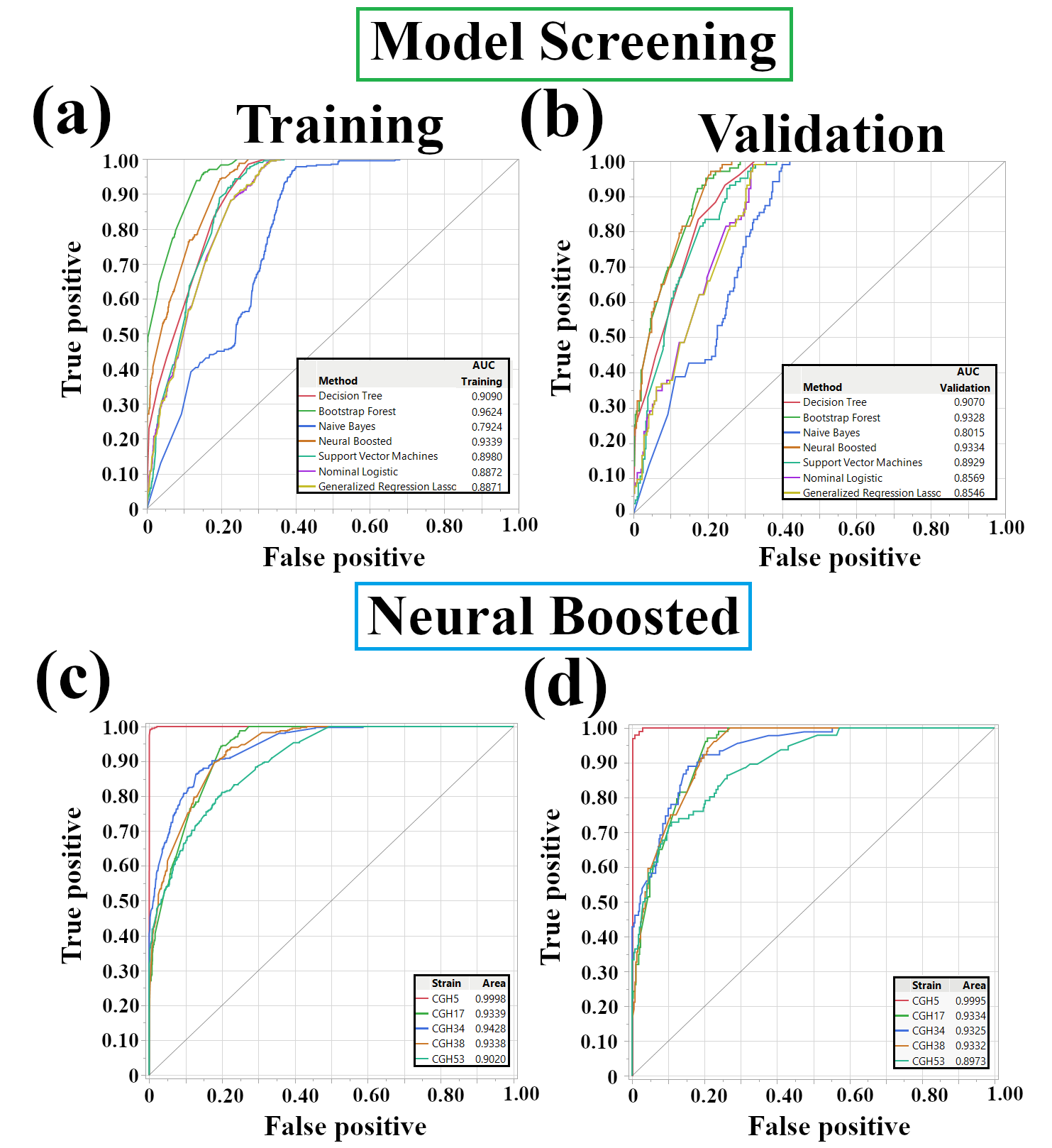

Supplement: Supplementary file 4 — Supplementary Material 4 [file 41598_2025_20277_MOESM4_ESM.zip › Fig. S2..tif]

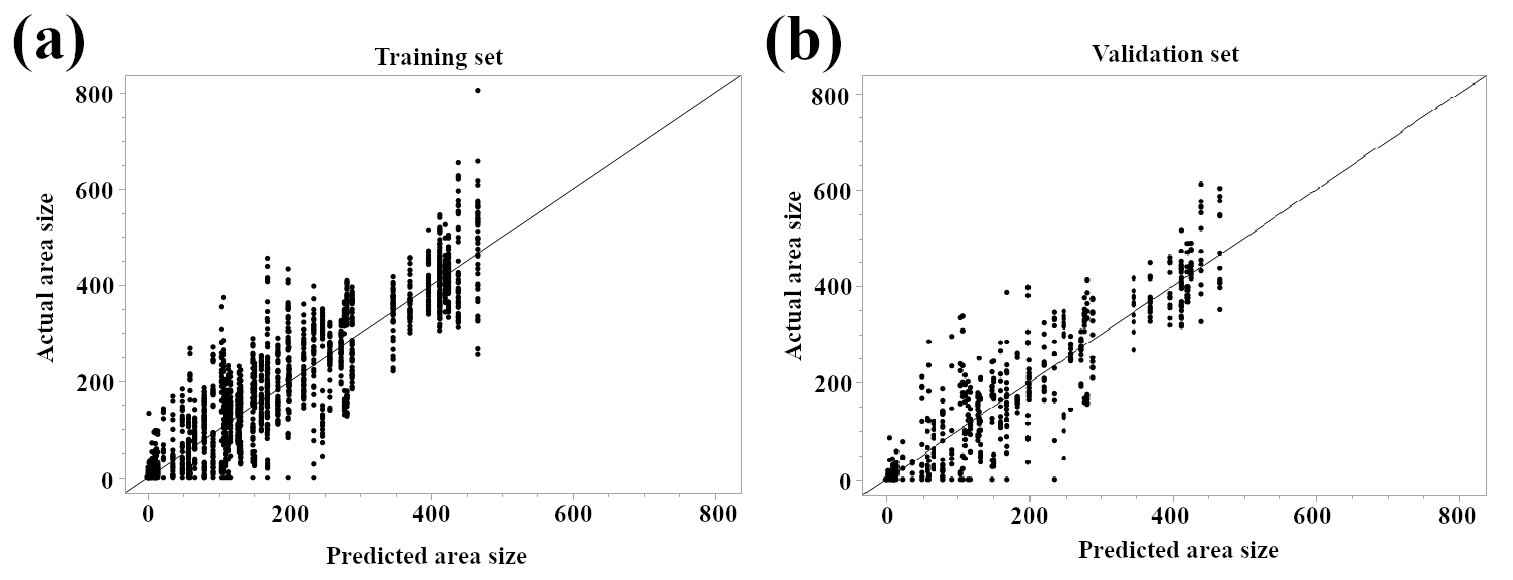

Supplement: Supplementary file 4 — Supplementary Material 4 [file 41598_2025_20277_MOESM4_ESM.zip › Fig. S3..tif]

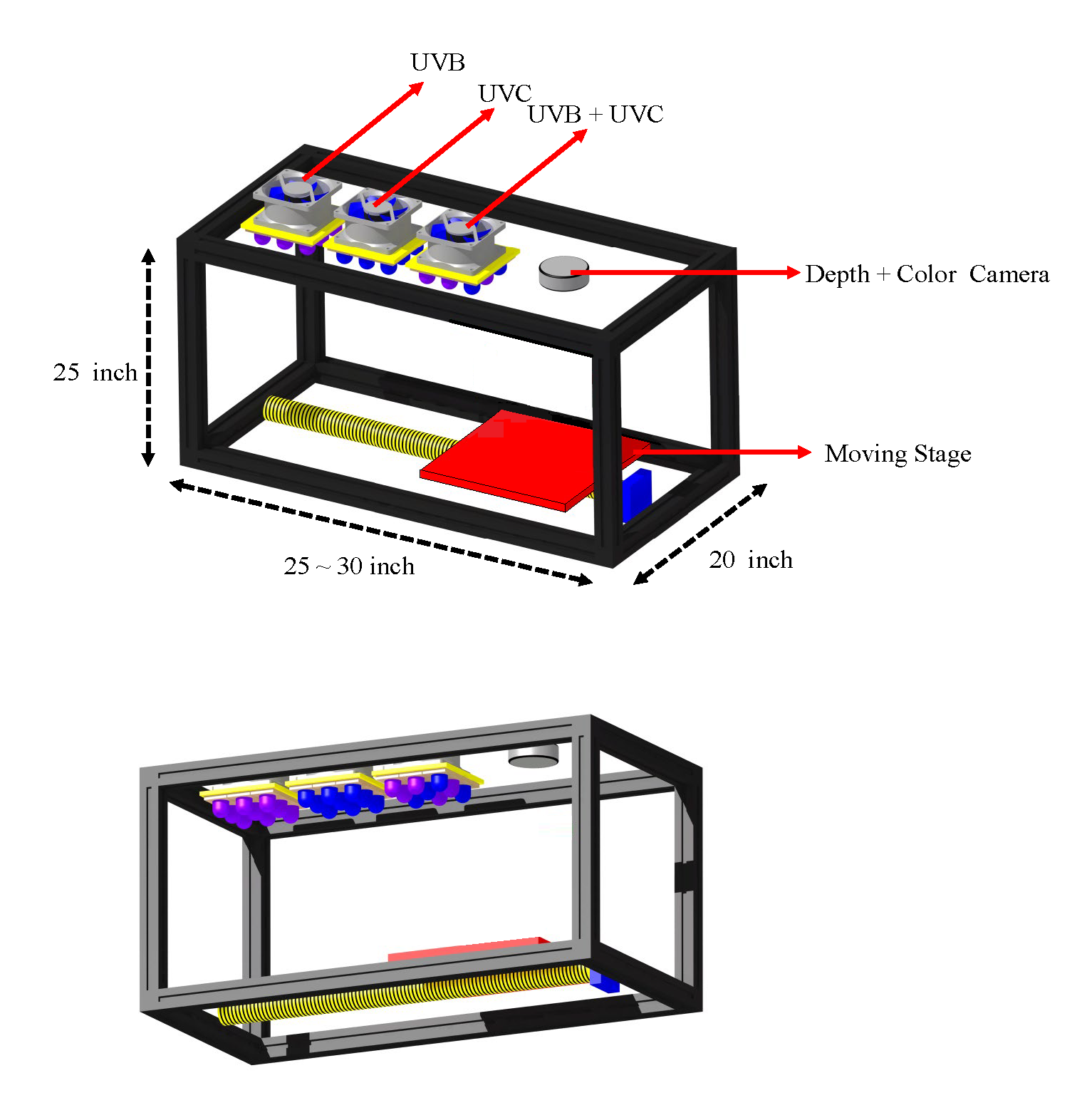

Supplement: Supplementary file 4 — Supplementary Material 4 [file 41598_2025_20277_MOESM4_ESM.zip › Fig. S4..tif]
